# Dietary cyanogen exposure and early child neurodevelopment: An observational study from the Democratic Republic of Congo

**DOI:** 10.1371/journal.pone.0193261

**Published:** 2018-04-17

**Authors:** Espérance Kashala-Abotnes, Marie-Thérèse Sombo, Daniel L. Okitundu, Marcel Kunyu, Guy Bumoko Makila-Mabe, Thorkild Tylleskär, Alla Sikorskii, Jean-Pierre Banea, Dieudonné Mumba Ngoyi, Désiré Tshala-Katumbay, Michael J. Boivin

**Affiliations:** 1 Centre for International Health, Department of Global Health and Primary Care, University of Bergen, Bergen, Norway; 2 Department of Neurology, University of Kinshasa, Congo-Kinshasa, Democratic Republic of the Congo; 3 Department of Psychiatry, Michigan State University, East Lansing, MI, United States of America; 4 National Nutrition Program, Ministry of Health, Congo-Kinshasa, Democratic Republic of the Congo; 5 School of Public Health, University of Kinshasa, Congo-Kinshasa, Democratic Republic of the Congo; 6 Department of Tropical Medicine, University of Kinshasa, Congo-Kinshasa, Democratic Republic of the Congo; 7 National Institute of Biomedical Research (INRB), Congo-Kinshasa, Democratic Republic of the Congo; 8 Department of Neurology, Oregon Health & Science University, Portland, OR, United States of America; 9 School of Public Health, Oregon Health & Science University, Portland, OR, United States of America; 10 Department of Neurology, Michigan State University, East Lansing, MI, United States of America; 11 Department of Ophthalmology, Michigan State University, East Lansing, MI, United States of America; Institute of medical research and medicinal plant studies, CAMEROON

## Abstract

**Background:**

Dietary cyanogen exposure from ingesting bitter (toxic) cassava as a main source of food in sub-Saharan Africa is related to neurological impairments in sub-Saharan Africa. We explored possible association with early child neurodevelopmental outcomes.

**Methods:**

We undertook a cross-sectional neurodevelopmental assessment of 12–48 month-old children using the Mullen Scale of Early Learning (MSEL) and the Gensini Gavito Scale (GGS). We used the Hopkins Symptoms Checklist-10 (HSCL-10) and Goldberg Depression Anxiety Scale (GDAS) to screen for symptoms of maternal depression-anxiety. We used the cyanogen content in household cassava flour and urinary thiocyanate (SCN) as biomarkers of dietary cyanogen exposure. We employed multivariable generalized linear models (GLM) with Gamma link function to determine predictors of early child neurodevelopmental outcomes.

**Results:**

The mean (*SD*) and median (IQR) of cyanogen content of cassava household flour were above the WHO cut-off points of 10 ppm (52.18 [32·79]) and 50 (30–50) ppm, respectively. Mean (*SD*) urinary levels of thiocyanate and median (IQR) were respectively 817·81 (474·59) and 688 (344–1032) μmole/l in mothers, and 617·49 (449·48) and 688 (344–688) μmole/l in children reflecting individual high levels as well as a community-wide cyanogenic exposure. The concentration of cyanide in cassava flour was significantly associated with early child neurodevelopment, motor development and cognitive ability as indicated by univariable linear regression (*p* < 0.05). After adjusting for biological and socioeconomic predictors at multivariable analyses, fine motor proficiency and child neurodevelopment remained the main predictors associated with the concentration of cyanide in cassava flour: coefficients of -0·08 to -.15 (*p* < 0·01). We also found a significant association between child linear growth, early child neurodevelopment, cognitive ability and motor development at both univariable and multivariable linear regression analyses coefficients of 1.44 to 7.31 (*p* < 0·01).

**Conclusion:**

Dietary cyanogen exposure is associated with early child neurodevelopment, cognitive abilities and motor development, even in the absence of clinically evident paralysis. There is a need for community-wide interventions for better cassava processing practices for detoxification, improved nutrition, and neuro-rehabilitation, all of which are essential for optimal development in exposed children.

## Introduction

The *Lancet* series on child development emphasised that more than 200 million children under five years of age are not fulfilling their developmental potential in low-income countries.[1] Children in low- and middle-income countries have a greater burden of risk factors for poor cognitive and behavioural development [2], of which exposure to food neurotoxicity is one [3]. More than 600 million people around the globe rely on bitter cyanogenic and potentially toxic cassava as their main staple food. In rural sub-Saharan Africa, the combination of high dependence on bitter cassava and insufficient cassava processing has been associated with outbreaks of konzo, a spastic paralysis in legs mainly affecting children and women of child-bearing age in ecologically degraded zones and poorer areas [4, 5]. Outbreaks of konzo mainly occur during times of food insecurity (seasonally in the lean season, or due to drought or food insecurity arising from displacement from war or conflict). In such situations, the processing of the cassava roots is often insufficient prior to food preparation and hence leads to dietary cyanogen exposure [6, 7]. Cassava roots and leaves are important sources of cyanide exposure, and the toxicity depends very much on the processing. Soaking of cassava roots for more than 72 hours reduced the cyanogen content by 90%, and boiling of cassava leaves in water with added palm oil reduced the cyanogen levels by 99% [8]. The main source of cyanogen exposure identified in the region has been from cassava roots [4] as cassava leaves are always cooked in boiled water with palm oil prior to consumption.

Konzo was initially characterized as a purely upper-motor neuron disease i.e. strictly confined to the motor pathways in the central nervous system (CNS) [9]. However, electrophysiological evidence suggests that there is a broader involvement of the CNS [9–11]. To date, there is no available data on the neurological effects of cyanogen exposure in children in affected areas, though a recent study from an affected area in the Democratic Republic of Congo (DRC) confirmed impaired neuro-cognition and motor proficiency in school-aged children [12].

None of the currently available research on cyanide exposure has studied very young children and the possible manifestations of neurological impairments in early childhood, even though WHO has estimated that 40% of foodborne disease burden (FBD) are found among children under five years of age. The highest burden is observed in Africa, and prevents children from reaching their full development potential [13]. We therefore aimed to explore whether dietary cyanogen exposure from bitter cassava in early childhood could be associated with the neurodevelopmental outcomes of very young children in Kahemba, DRC.

## Subjects and methods

This paper was prepared according to the STROBE guidelines for reporting of observational studies [14] (S1 File).

### Study site

The study was conducted in Kahemba, which is a severely konzo-affected zone in the Bandundu province located south-west in the DRC, bordering Angola. Kahemba has an area of 20 000 km^2^ and an estimated population of 250 000 inhabitants relying mostly on subsistence cassava farming. Bitter cassava is the staple crop grown, and it is processed for food consumption by women. The processing consists of soaking the cassava roots in water for a recommended period of three to four nights, then drying them in the sun for one to two days before pounding them to make flour. The flour is subsequently mixed with boiling water to make a soft dough, which is eaten with gravy. In times of intense cassava trading and/or agro-ecological crises such as drought, shortcuts in cassava processing are common [7, 15], and signs of intoxication occur as residual amounts of cyanogenic compounds are left in the roots [16]. Over the last decades, Kahemba has been the scene of repeated outbreaks of konzo and its prevalence in certain villages is reportedly up to 20% [15, 17].

### Study design and participants

We carried out a cross-sectional study within the cohort of the longitudinal study (parent study) on the neuropsychological effects of cassava in school-aged children [12]. From this parent study, we have recruited households (mothers/caretakers) with 12–48 month old children with and without konzo that have consented to participate. Children with a medical history of illnesses possibly affecting the CNS, such as epilepsy, cerebral palsy, and acute malnutrition, were excluded from the study. After consent, we were able to enrol recruit a convenient sample of 61 and 53 households with and without konzo, respectively. At the time of the study, none of the eligible children was listed as being a konzo subject in the health zone incidence registry.

### Study procedure

#### Sociodemographic, economic and home environment covariates

Local health workers conducted structured interviews with the mothers/caregivers to gather information on sociodemographic, socioeconomic, and home environment during home visits. Parental level of education was scaled from 0 (no education) to 4 (university level). Socioeconomic status was ranked using a generated wealth index based on assets, quality of housing (type of floor, roofing, toilet facilities, water source, electricity, etc.). This method has been used previously as a proxy in the same setting [12]. The short version of the Caldwell Home Observation for Measurement of the Environment (HOME) (S1 Table), which has been adapted for the African context [18], was used to assess parenting style and the child’s level of stimulation and the learning opportunities offered by the home environment. This tool has been previously used in the DRC and have been shown to be a useful measure of mother-child interaction [12]. We only conducted mothers/caregivers 18 items interviews without any home observation due to financial and time constraints.

#### Child’s and mother’s health covariates

All children were systematically screened for signs of the disease through a clinical examination that included a neurological examination. Anthropometric measurements were taken according to standard procedures [19]. Mid-upper arm circumference (MUAC) was used as a standard measurement of nutritional status according to WHO recommendation for children 6 to 60 months of age. The cut-off value of 115 mm indicates severe acute malnutrition [20, 21]. Anthropometrics Z-scores and body mass index-for-age were calculated and used as continuous variables.

Maternal depression and anxiety symptoms were assessed through a structured interview using Hopkins symptoms checklist-10 (HSCL-10) and Goldberg Depressive Anxiety Scale (GDAS) (S1 Table). HSCL-25 is a well-known and widely used screening instrument [22]. We used the short version HSCL-10 [23], which is considered a good screening instrument in primary health care settings, and research [24, 25]. GDAS is an easy to administer scale [26] that has been validated in the DRC [27]. All derived scores from HSCL-10 and GDAS were analysed as continuous variables.

#### Measure of early child neurodevelopment and neurocognitive outcomes

Early child development and cognition were measured through clinical observation and interviews with caregivers/parents. The tools selected to measure early child development and cognition in the present study, are presented in S1 Table.

The Mullen Scales of Early Learning (MSEL) [28] is easy to administer and has been found useful for assessing child development and cognition in low resource settings. It is a quick and reliable assessment tool for childhood development measuring cognitive ability and motor development [28]. It has been translated into French [18]. The study team was trained by a psychologist (MJB) and MSEL instructions were administered in the local language.

The Gensini Gavito Scale (GGS) is specifically validated in DRC, and was used to cross-validate findings on early child neurodevelopment, cognitive ability and motor development outcomes. It is a neurodevelopmental scale developed for the assessment of psychomotor development and growth of children in low-income settings [29]. The scale is widely used and has been adapted locally.

The Ten Questions Questionnaire (TQQ) [30] is a screening tool for child disability that has been developed for use in resource-limited settings, and was used to gather information on child development and disabilities as perceived by the mother (S1 Table). It is the most widely used tool for child disability assessment in low- and middle-income countries [31, 32].

At the local health station, three neuropsychiatrists who did not have access to the health zone registry of konzo patients received the mother-child dyad. They performed the clinical evaluation of the child, and gathered all medical information from pregnancy, milestones, growth, and breastfeeding practices. For quality assurance purposes, two doctors performed the same tasks, and one conducted the parent interview. Each child was evaluated according to a standardised format in the same setting and in presence of the child’s primary caregiver.

#### Assessment of dietary cyanogen exposure

The sample collection and storage methods have been previously described by colleagues [33]. Briefly, a team of laboratory technicians collected samples of urine and cassava flour in each household on the day of the clinical examination of the child, and measured the concentration using the SCN picrate kit D1 and B2 protocols [34]. Mothers were given instructions on how to collect the child’s urine using a clean jar for younger children. In the present study, we only measured the cyanide-yielding capacity in household cassava flour, and thiocyanate (SCN, metabolite of cyanide) in urine from the mother-child dyads as the sole source of exposure. Exposure from cassava leaves is unlikely or minimal due to the cooking process prior to the consumption of cassava leaves. Protocols to determine the total cyanide-yielding capacity in cassava products (Kit B2) and thiocyanate in urine (Kit D1) are available on line:

(http://biology-assets.anu.edu.au/hosted_sites/CCDN/five.html).

### Statistical analyses

Early child development, cognitive ability and motor development were the main outcomes, whereas dietary cyanogen was the main exposure variable considered in the analysis.

Means, medians and interquartile ranges were used to summarize the distributions of continuous variables, whereas proportions were calculated for dichotomous variables. Linear regression, generalized linear model (GLM) analyses with Gamma link function were used for univariable and multivariable adjusted analyses. Linear regression was used to explore associations between MSEL and GGS scores, and dietary cyanogen concentration in household cassava flour and urine. Regression models first examined each explanatory variable for association with the main outcomes, with regression parameters providing unadjusted estimates of the association. Second, multivariable adjusted models were built to include the main exposure variables while adjusting for relevant sociodemographic and biological factors: age, sex, child’s anthropometric characteristics, the HOME score, socioeconomic status, maternal depression/anxiety, child nutritional status, and maternal risk factors during pregnancy. Main outcomes and exposure variables were analyzed as continuous variables. Other predictors such as maternal depression/anxiety and child nutritional status were also analysed as continuous variables. Data concerning maternal smoking and alcohol consumption during pregnancy, as well as current information including introduction of solid food before six months of age, and the presence of previous morbidities such as epilepsy, sickle cell anemia, or cerebral palsy in the child were analyzed as dichotomous variables. Limited backward elimination was performed: explanatory variables that were not significant were removed from the models only when they did not change the estimates of other effects. Lastly, we dichotomized the household variable in two groups of konzo-affected and unaffected households to explore whether there were differences between the two groups. All tests were 2-tailed and conducted at 0·05 level of significance. Analyses were done with the statistical package Stata 14 (www.stata.com).

### Ethical approval

The Institutional Review Board of the Ministry of Health in DRC number CE/368/2014 approved the study. Signed or fingerprint informed consent was obtained from each mother (caregiver) / guardian.

## Results

### Sample characteristics

A total of 114 mother-child dyads were included in the study. Overall, the children’s mean age (*SD*) was 31 (4) months old (minimum: 21 and maximum: 42 months). They were 64 (56%) boys and 50 (44%) girls with no gender differences observed (95% CI: 0·5–2·0). The majority of children, 99 (87%), were born in the region and all children included in the study resided in Kahemba for more than six months prior to the study.

In terms of households, 61 (53·5%) children belonged to konzo-affected households, whereas 53 (46·5%) to unaffected households. Of the 15 (13%) born outside, the majority 11 (73·3%) belonged to konzo unaffected households, whereas 4 (26·7%) to konzo-affected households (OR: 3·7 95% CI: 1·1–12·5).

### Sociodemographic, socioeconomic and home environment characteristics

These characteristics are presented in Tables 1 and 2. The majority of parents had a secondary level of education, and more than half of children were weaned before six months of age. Consumption of alcohol and smoking were not common during pregnancy and at the time of the study visit among the studied population. A good proportion of parents used stimulation and learning opportunities such as storytelling, and homemade toys for their kids. Spanking and scolding were the common mode of punishment (Table 2). While comparing children based on the household konzo status, i.e. households with konzo-affected subjects vs. unaffected households, children from konzo-affected and unaffected households had similar sociodemographic characteristics, though significant differences were noticed in the family wealth index, age of introduction to solid foods, and maternal characteristics (Table 1).

**Table 1:** Demographic, maternal, and child characteristics of the 114 studied children aged 12–48 months in Kahemba, Democratic Republic of Congo.

	Total		HH with konzo	HH without konzo
Variables (Units)	Mean (*SD*)	Median (IQR)	Mean (*SD*)	Mean (*SD*)
***Sociodemographic***				
Child’s age (months)	31.0 (4.2)	31 (29–34)	31.5 (4.5)	30.4 (3.9)
Mother age at birth (years)	29.7 (9.3)	28 (22–37)	30.8 (10.0)	28.5 (8.3)
Father age at birth (years)	38.5 (13.6)	37.5 (26.5–48.5)	38.1 (14.5)	39.1 (12.7)
Household size (*n*)	7.7 (2.8)	7 (6–9)	7.7 (2.9)	7.6 (2.7)
Children ≤ 17years old in the house (*n*)	4.6 (2.1)	4 (3–6)	4.5 (1.9)	4.8 (2.4)
Number of adults > 18 years old in the house	3.0 (1.6)	2 (2–4)	3.2 (1.9)	2.7 (1.3)
Number of sibling (*n*)	4.1 (2.5)	4 (2–6)	4.3 (2.7)	3.9 (2.2)
Rank of the child (*n*)	3.9 (2.5)	3 (2–6)	4.1 (2.7)	3.7 (2.2)
Monthly income (Congo Francs)	88336.6 (32.8)	50 (30–50)	84721 (45219)	92508 (50600)
Wealth index (scores)	2.5 (2.5)	2 (0–3)	1.74 (1.5)	3.31 (3.0) **
***Maternal***				
Hopkins Symptoms Check List (scores)	7.98 (6.38)	7 (2–13)	9.8 (6.8)	5.9 (5.2) **
Goldberg Depression Anxiety Scale (scores)	7.4 (5.6)	7 (1–13)	8.5 (5.7)	6.0 (5.2)*
Mother weight (kg)	48.2 (8.0)	47 (43–53)	46.7 (8.2)	49.9 (7.4)*
Mother height (cm)	154.5 (6.3)	154 (150–159)	153.9 (6.6)	155.7 (5.8)
***Child***				
Birthweight (gram)	3409.3 (655.9)	3500 (3000–3800)	3362 (649)	3462 (665)
Child’s weaning age (months)	5.5 (3.0)	5 (3–7)	5.00 (3.2)	6.13 (2.6)*
Ten questions (scores)	0.5 (1.0)	0 (0–1)	0.4 (1.0)	0.6 (1.1)
Child weight (kg)	10.9 (2.2)	11 (9–12)	10.9 (1.9)	10.8 (2.6)
Height (cm)	84.8 (5.6)	85 (81–88)	85.5 (5.3)	84.0 (5.9)
Mid upper arm circumference (cm)	14.7 (1.3)	15 (14–15)	14.7 (1.1)	14.8 (1.4)
Weight for age *z*-score	-1.7 (1.6)	-1.71 (-(2.64 to -0.7)	-1.7 (1.3)	-1.8 (1.8)
Height for age *z*-score	-2.1 (1.4)	-1.98 (- 2.99 to -1.04)	-1.9 (1.4)	-2.2 (1.5)
Head circumference (cm)	48.3 (1.8)	48 (47–49)	48.4 (1.7)	48.1 (1.8)
Number of meals the previous day (*n*)	1.98 (0.6)	2 (2–2)	2.0 (0.6)	1.9(0.6)

*T-test *p*-value < 0.05

** *T*-test *p*-value < 0.01

HH: households. Konzo: irreversible upper-motor disease linked to cyanide neurotoxicity from poorly processed cassava

**Table 2:** Proportion of different factors related to the child’s medical and perinatal history, parental level of education, stimulation & learning opportunities for the 114 studied children aged 12–48 months in Kahemba, Democratic Republic of Congo.

	Total	HH with konzo	HH without konzo
Variables	*N* (%)	*N* (%)	*N* (%)
**Child’s factors**			
Household with konzo* affected member	61 (54)	61 (100)	00 (00)*
Child’s previous morbidities #	18 (15.8)	12 (19.7)	06 (11.3)
Introduction of solid foods < 6 months	61 (55)	41 (68.3)	20 (38.5) **
Maternal smoking during pregnancy	01 (01)	01 (0.7)	00 (0)
Maternal alcohol during pregnancy	32 (28)	20 (33.3)	12 (23.1)
Number of nights of soaking of cassava < 3 days	24 (21.4)	12 (20)	12 (23.1)
Number of nights of soaking of cassava ≥ 3 days	88 (78.6)	48 (80)	40 (76.9)
Mother level of education*			
*Primary*	39 (35)	29 (48.3)	10 (18.9)
*Secondary*	57 (50)	26 (43.3)	31 (58.5)
*Professional*	04 (3.5)	01 (1.7)	03 (5.7)
*None*	13 (12)	04 (6.7)	09 (16.98)
**Father level of education** (*N* = 110)			
*Primary*	16 (15)	12 (20.3)	04 (6.8)
*Secondary*	76 (69)	42 (71.2)	34 (66.7)
*Professional*	07 (06)	01 (1.7)	06 (11.8)
*University*	07 (06)	03 (5.1)	04 (7.8)
*None*	04 (03)	01 (1.7)	03 (5.9)
**Stimulation, learning opportunities & parenting style**			
Storytelling to kids	28 (25)	16 (26.7)	12 (23.1)
Uses books with pictures	32 (28.6)	17 (23.3)	15 (28.5)
Read to the child	26 (23.2)	15 (25)	11 (21.2)
Play with kitchenette	72 (64.3)	38 (63.3)	34 (65.4)
Play with homemade toys	82 (73.2)	42 (70)	40 (76.9)
Play with bought toys	43 (38.4)	20 (33.3)	23 (44.2)
Left alone home more than 10 times/month	05 (04.5)	01 (1.7)	04 (7.7)
Explain without being upset	43 (38.4)	24 (40)	19 (36.5)
Raise voice	92 (82.1)	50 (83.3)	42 (80.8)
Take away toys to punish	35 (31.3)	19 (31.7)	16 (30.8)
Give extra work to punish	25 (22.3)	12 (20)	13 (25)
Spank to punish	64 (57.1)	38 (63.3)	26 (50)
Use stick to punish	44 (39.3)	23 (38.3)	21 (40.4)
Hit on the head to punish	14 (12.5)	04 (6.7)	10 (19.2)*

HH: household; Konzo: irreversible upper-motor disease linked to cyanide neurotoxicity from poorly processed cassava.

#Child previous morbidities included convulsions, brain trauma, sickle cell anemia, or central nervous system infections.

*Chi-square *p*-value < 0.05.

**Chi-square *p*-value < 0.01.

### Child and maternal health

Child and maternal health characteristics are presented on Table 1.

In general, the majority of children did not show any clinical signs of the disease at the screening clinical and neurological examination. However, although none of the children were listed as konzo patients in the incidence list of the health zone registry at the time of the study, we were able to detect/screen neurological signs of the disease among three of the children. Those three children came from konzo-affected households and the observed neurological signs were symmetric spastic abnormality of gait (two of the three children were unable to walk unsupported), bilaterally exaggerated knee and/or ankle reflexes and/or jerk reflexes, Babinski reflexes, language difficulties (two of the children), and nystagmus (one child). These neurological signs are suggestive of moderate to severe degree of the disease. We were also able to obtain the urinary thiocyanate concentration from one child only (688 μmole/litre), but unfortunately the urine samples of the two other children were not available. All three children were born in Kahemba, and aged 27, 32, and 41 months, respectively, at the time of the clinical examination. Their medical history and background were similar to that of the whole group of children.

### Early child neurodevelopment and neurocognitive outcomes

Subscales scores of MSEL and GGS are shown on Table 3. No group differences were noticed in terms of early child development and neurocognitive outcomes, though children in the konzo-affected households had a slight tendency towards lower scores in gross motor (Table 3).

**Table 3:** Mullen Scale for Early Learning and Gensini Gavito Scale scores of the 114 studied children aged 12–48 months old in Kahemba, Democratic Republic of Congo.

	HH with konzo	HH without konzo	Total
Variables	(*N* = 61)	(*N* = 53)	(*N* = 114)
Mullen Scale for Early Learning			
T scores Gross motor			
Mean (*SD*)	38.5 (11.5)	43.6 (10.6) #	41.09 (11.30)
Median (IQ)	38.0	45	42
Median (IQR)	32–46	38–50	(33–50)
T score visual reception			
Mean (*SD*)	35.8 (11.1)	39.5 (11.3)	37.41 (11.26)
Median (IQ)	35	41	36
Median (IQR)	28–44	30–48	29–45
T scores fine motor			
Mean (*SD*)	35.4 (12.3)	39.4 (13.9)	37.19 (13.15)
Median (IQ)	36	39	38
Median (IQR)	25–42	32–46.5	27–43
T scores receptive language			
Mean	41.2 (11.5)	42.5 (8.6)	41.76 (10.26)
Median (IQ)	41	44	41
Median (IQR)	33–49	35–51	34–49
T scores expressive language			
Mean (*SD*)	38.0 (9.9)	38.8 (9.8)	38.4 (9.8)
Median (IQ)	35	38	36
Median (IQR)	30.5–43	30–44	30–43
T scores cognition			
Mean (*SD*)	151.3 (35.9)	160.7 (33.8)	155.5 (35.1)
Median (IQ)	147.5	167	153
Median (IQR)	125.5–174.5	130–188	129–182
Early Learning Composite			
Mean (*SD*)	77.3 (16.6)	82.1 (15.0)	79.4 (16.0)
Median (IQ)	75	84	77
Median (IQR)	65.5–87.5	70–94	67–91
Gensini Gavito Scale			
Child developmental age (month)			
Mean (*SD*)	28.6 (5.8)	26.6 (5.4) #	27.7 (5.7)
Median	30	27	30
Median (IQR)	24–30	24–30	24–30
Child developmental quotient (%)			
Mean (*SD*)	91.2 (15.4)	88.7 (15.3)	90.0(15.3)
Median (IQ)	94	91	91
Median (IQR)	83–100	77–100	77–100

HH: households; Konzo: irreversible upper-motor disease linked to cyanide neurotoxicity from poorly processed cassava. TSGM: t-core gross motor, TSVR: t-score visual reception, TSFM: t-score fine motor, TSRL: t-score receptive language, TSEL: t-score expressive language.

^#^*P*-value = 0.06 at *t*-test or Mann-Whitney test for mean or median comparisons, respectively. IQR: interquartile ranges (25–75%).

A total of 9 children (4 from konzo-affected households, and 5 from unaffected households) refused to perform neurocognitive tasks. They were similar to the tested children in all other characteristics, but had a significantly lower developmental age on the GGS assessment (*p* < 0·02).

### Assessment of dietary cyanogen exposure

Table 4 summarizes the overall mean (*SD*) and median (Inter Quartile Range: IQR) values of cyanogen exposure. The cyanogen content in cassava flour was above the safe limit of 10 ppm [35] and the level of SCN urinary excretion was above 350 μmole/litre in both affected and unaffected households. There was no significant difference between the groups (*t*-test *p*-value > 0.05).

**Table 4:** Dietary cyanogen cassava content and urinary thiocyanate excretion for the studied 114 children aged 12–48 months in Kahemba, Democratic Republic of Congo.

	HH with konzo	HH without konzo	Total	
Variables	Mean (*SD*)	Mean (*SD*)	Mean (*SD*)	Median (IQR)
Thiocyanate in child’s urine (μmole/liter)	622.1 (481.0)	612.5 (417.2)	617.49 (449.48)	688 (344–688)
Thiocyanate in mother’s urine (μmole/liter)	843.1 (512.8)	788.5 (430.0)	817.81 (474.59)	688 (344–1032)
Cyanogen in cassava flour (ppm)	53.5 (34.3)	50.7 (31.2)	52.18 (32.79)	50 (30–50)

HH: households; Konzo: irreversible upper-motor disease linked to cyanide neurotoxicity from poorly processed cassava. IQR: Inter quartile range (25–75%).

### Association between early child outcomes, dietary cyanogen exposure and other covariates

Univariable linear regression analyses showed an association between early child development (GGS), cognitive ability and motor development (MSEL) outcomes, and parental level of education, monthly income, child’s anthropometrics, and the cyanogen content in cassava flour (Table 5). There was no significant association between maternal depression/anxiety, home environment or parenting style in the univariable linear regression analysis (*p*-value > 0·05) (Table 5).

**Table 5:** Overall and by household groups crude coefficient (*p*-value) from the univariable linear regression for scores derived from Mullen Scale of Early Learning, Gensini Gavito Scale, and cassava cyanogen content for the 114 studied children aged 12–48 months in Kahemba, democratic republic of Congo.

	ELC	GM	FM	VR	RL	ELA	CDA	PDQ	CF
Sociodemographic									
Child’s age (months)									
Overall	0.40 (0.34)	-.33 (0.49)	0.08 (0.80)	-.11 (0.70)	0.08 (0.74)	**0.52 (0.04)**	**0.66 (0.00)**	**-.66 (0.06)** **#**	0.20 (0.79)
HH with konzo	0.37 (0.50)	-.47 (0.52)	0.08 (0.83)	-.08 (0.82)	0.14 (0.71)	0.36 (0.27)	0.74 (0.00)	-.52 (0.28)	0.81 (0.746
HH without konzo	0.70 (0.28)	-.29 (0.65)	0.29 (0.64)	0.06 (0.92)	0.07 (0.86)	**0.73 (0.04)**	**0.50 (0.01)**	-.96 (0.08)	-0.91 (0.47)
Child’s gender									
Overall	0.32 (0.92)	0.37 (0.89)	-2.42 (0.37)	0.88 (0.71)	0.29 (0.88)	3.07 (0.13)	0.07 (0.95)	1.69 (0.57)	-.38 (0.95)
HH with konzo	-4.84 (0.30)	-3.57 (0.38)	-6.01 (0.07)	-2.17 (0.48)	-2.76 (0.39)	2.58 (0.35)	-0.46 (0.77)	0.19 (0.96)	7.12 (0.46)
HH without konzo	6.59 (0.16)	3.62 (0.33)	1.91 (0.66)	4.56 (0.20)	4.03 (0.13)	3.67 (0.22)	0.60 (0.69)	3.28 (0.45)	-9.18 (0.33)
Father’s education									
Overall	**4.43 (0.03)**	2.70 (0.09)	2.57 (0.12)	1.31 (0.35)	**2.83 (0.03)**	**2.67 (0.03)**	0.29 (0.68)	1.38 (0.47)	-6.53(0.13)
HH with konzo	**10.43 (0.00)**	**5.59 (0.03)**	**6.33 (0.01)**	**3.45 (0.08)**	**5.82 (0.01)**	**5.00 (0.00)**	1.20 (0.29)	2.53 (0.40)	-10.19(0.14)
HH without konzo	-.37 (0.88)	0.11 (0.95)	-.58 (0.86)	-1.74 (0.44)	0.59 (0.68)	0.96 (0.61)	-0.13 (0.88)	0.88 (0.72)	-3.44(0.55)
Mother’s education									
Overall	3.16 (0.16)	**3.83 (0.03)**	0.31 (0.87)	1.06 (0.51)	2.03 (0.16)	**2.93 (0.03)**	0.54 (0.47)	1.73 (0.39)	-2.33(0.59)
HH with konzo	5.94 (0.12)	4.48 (0.18)	1.11 (0.69)	2.68 (0.28)	3.63 (0.17)	2.97 (0.17)	0.13 (0.92)	-1.28 (0.69)	-1.59(0.83)
HH without konzo	0.57 (0.84)	2.67 (0.21)	-1.02 (0.68)	-.68 (0.74)	0.87 (0.56)	2.94 (0.07)	1.06 (0.25)	4.11 (0.11)	-2.66(0.62)
Wealth index (scores)									
Overall	0.52 (0.43)	0.85 (0.09)	-0.08 (0.87)	0.69 (0.13)	0.46 (0.28)	0.49 (0.23)	0.11 (0.63)	0.85 (0.16)	-.91 (0.49)
HH with konzo	-.66 (0.70)	-.56 (0.75)	-1.51 (0.24)	-.07 (0.95)	0.44 (0.70)	0.11 (0.92)	0.42 (0.43)	1.24 (0.37)	-.99 (0.75)
HH without konzo	0.48 (0.51)	0.71 (0.20)	-.17 (0.79)	0.57 (0.29)	0.44 (0.31)	0.73 (0.15)	0.20 (0.42)	1.02 (0.14)	-.85 (0.57)
Monthly income									
Overall	0.00 (0.15)	0.00 (0.11)	**0.00 (0.03)**	0.00 (0.13)	-0.03 (0.90)	0.00 (0.28)	-4.75 (0.68)	0.00 (0.53)	-.00 (0.87)
HH with konzo	0.00 (0.24)	0.00 (0.28)	0.00 (0.10)	0.00 (0.39)	0.00 (0.80)	0.00 (0.22)	-0.00 (0.27)	8.75 (0.85)	-.00 (0.66)
HH without konzo	0.00 (0.57)	0.00 (0.45)	0.00 (0.25)	0.00 (0.36)	-0.00 (0.50)	0.00 (0.67)	0.00 (0.47)	0.00 (0.44)	0.00 (0.77)
Maternal depression/anxiety symptoms									
GDAS (scores)									
Overall	0.07 (0.80)	-0.14 (0.57)	0.20 (0.38)	0.11 (0.59)	-0.04 (0.82)	-0-05 (0.77)	-.14 (0.16)	-.39 (0.14)	0.29 (0.64)
HH with konzo	0.29 (0.46)	0.01 (0.98)	0.38 (0.20)	0.32 (0.24)	-0.01 (0.97)	0.03 (0.89)	-.18 (0.18)	-.25 (0.49)	0.25 (0.76)
HH without konzo	-.02 (0.97)	-.02 (0.95)	0.15 (0.70)	0.01 (0.97)	-0.03 (0.90)	-0.12 (0.69)	-.20 (0.19)	-.79 (0.06)	0.23 (0.81)
HSCL-10 (scores)									
Overall	-0.21 (0.39)	-0.15 (0.48)	-0.02 (0.88)	-0.07 (0.71)	-0.23 (0.16)	-0.22 (0.16)	-.06 (0.49)	-.18 (0.45)	0.24 (0.66)
HH with konzo	0.03 (0.92)	0.14 (0.61)	0.17 (0.49)	0.22 (0.33)	-0.19 (0.43)	-0.14 (0.45)	-.09 (0.43)	-.09 (0.76)	-0.15 (0.83)
HH without konzo	-.48 (0.29)	-.30 (0.48)	-.19 (0.60)	-.44 (0.15)	-0.32 (0.22)	-0.37 (0.19)	-.18 (0.24)	**-.98 (0.02)**	0.93 (0.34)
Child’s early neurodevelopment									
CDA (months)									
Overall	**1.01 (0.00)**	**0.55 (0.06)** **#**	**0.71 (0.00)**	**0.50 (0.02)**	**0.68 (0.00)**	**0.59 (0.00)**	1	**2.02 (0.00)**	**-1.26 (0.04)**
HH with konzo	**0.93 (0.02)**	0.50 (0.25)	**0.68 (0.02)**	**0.59 (0.04)**	**0.67 (0.02)**	0.43 (0.09)	1	**1.95 (0.00)**	-1.36 (0.11)
HH without konzo	**1.30 (0.00)**	**0.69 (0.08)**	**0.86 (0.02)**	0.68 (0.07)	**0.74 (0.01)**	**0.81 (0.00)**	1	**2.16 (0.00)**	-1.36 (0.13)
PDQ (%)									
Overall	**0.40 (0.00)**	**0.31 (0.00)**	**0.28 (0.00)**	**0.27 (0.00)**	**0.29 (0.00)**	**0.14 (0.04)**	**0.28 (0.00)**	1	**-.45 (0.04)**
HH with konzo	**0.36 (0.02)**	**0.29 (0.04)**	**0.26 (0.00)**	**0.26 (0.00)**	**0.26 (0.01)**	0.10 (0.31)	**0.28 (0.00)**	1	**-.68 (0.03)**
HH without konzo	**0.48 (0.00)**	**0.39 (0.00)**	**0.31 (0.02)**	**0.29 (0.03)**	**0.34 (0.00)**	**0.21 (0.06)**	**0.27 (0.00)**	1	-.18 (0.56)
Child’s weaning age (months)									
Overall	0.51 (0.34)	0.29 (0.47)	0.29 (0.49)	0.27 (0.47)	0.52 (0.12)	0.18 (0.58)	-.04 (0.82)	0.28 (0.56)	1.27 (0.24)
HH with konzo	0.41 (0.55)	0.29 (0.59)	0.21 (0.69)	0.16 (0.73)	0.44 (0.35)	0.15 (0.74)	-.08 (0.74)	0.14 (0.83)	**3.05 (0.03)**
HH without konzo	0.41 (0.65)	-.10 (0.87)	0.26 (0.78)	0.21 (0.75)	0.63 (0.22)	0.29 (0.66)	-.08 (0.76)	0.82 (0.34)	-2.05 (0.26)
Introduction of solid food <6 months									
Overall	3.30 (0.33)	1.12 (0.69)	2.46 (0.37)	1.50 (0.52)	2.72 (0.20)	1.22 (0.55)	-.38 (0.73)	1.88 (0.53)	-3.09 (0.64)
HH with konzo	-.59 (0.90)	0.21 (0.96)	1.41 (0.69)	-1.89 (0.55)	1.79 (0.59)	-1.14 (0.69)	0.13 (0.94)	0.23 (0.96)	-1.83 (0.85)
HH without konzo	5.93 (0.22)	-1.06 (0.79)	1.89 (0.67)	3.76 (0.29)	3.45 (0.21)	3.98 (0.17)	0.31 (0.84)	5.54 (0.21)	-2.59 (0.79)
Child’s anthropometry									
Weight-for-age *z* scores									
Overall	**3.84 (0.00)**	**3.24 (0.00)**	**2.73 (0.00)**	**1.91 (0.02)**	**2.59 (0.00)**	**2.30 (0.00)**	**1.53 (0.00)**	**4.18 (0.00)**	-3.29 (0.12)
HH with konzo	**3.59 (0.04)**	**3.76 (0.01)**	**2.23 (0.04)**	1.84 (0.12)	**2.82 (0.01)**	**2.03 (0.05)**	**1.49 (0.01)**	**5.04 (0.00)**	-3.64 (0.31)
HH without konzo	**3.82 (0.00)**	**2.874 (0.01)**	**3.08 (0.00)**	1.79 (0.10)	**2.59 (0.00)**	**2.35 (0.00)**	**1.51 (0.00)**	**3.65 (0.00)**	-3.19 (0.23)
Height-for-age *z* scores									
Overall	**7.17 (0.00)**	**4.77 (0.00)**	**3.78 (0.00)**	**4.33 (0.00)**	**4.36 (0.00)**	**3.20 (0.00)**	**2.03 (0.00)**	**6.03 (0.00)**	-2.93 (0.22)
HH with konzo	**6.87 (0.00)**	**4.65 (0.00)**	**2.22 (0.02)**	**3.70 (0.00)**	**4.87 (0.00)**	**3.55 (0.00)**	**1.99 (0.00)**	**7.56 (0.00)**	-.42 (0.91)
HH without konzo	**7.31 (0.00)**	**4.98 (0.00)**	**4.39 (0.00)**	**4.06 (0.00)**	**4.36 (0.00)**	**3.76 (0.00)**	**1.99 (0.00)**	**4.77 (0.00)**	-5.54 (0.08)
Cyanogenic exposure									
SCN in child’s urine (μmole/liter)									
Overall	-0.00 (0.53)	0.00 (0.52)	-0.00 (0.53)	-0.00 (0.94)	-0.00 (0.55)	-0.00 (0.63)	**-.00 (0.04)**	-.00(0.44)	0.00 (0.52)
HH with konzo	-0.00 (0.21)	0.00 (0.97)	**-0.01 (0.02)**	-0.00 (0.87)	-0.00 (0.27)	-0.00 (0.56)	-.00 (0.10)	-.00(0.51)	0.01 (0.21)
HH without konzo	0.00 (0.47)	0.00 (0.22)	0.00 (0.33)	0.00 (0.88)	0.00 (0.47)	-0.00 (0.98)	-.00 (0.29)	-.00(0.71)	-0.01 (0.58)
SCN in mother’s urine (μmole/liter)									
Overall	-0.01 (0.15)	-0.00 (0.30)	-0.00 (0.21)	-0.00 (0.20)	-0.00 (0.39)	-0.00 (0.58)	-.00 (0.19)	-.00 (0.60)	0.00 (0.91)
HH with konzo	-0.00 (0.32)	-0.00 (0.49)	-.00 (0.71)	-0.00 (0.29)	-0.00 (0.49)	-0.00 (0.55)	-.00 (0.14)	-.00 (0.39)	-0.00 (0.79)
HH without konzo	-0.00 (0.40)	-0.00 (0.75)	-0.00 (0.26)	-0.00 (0.71)	-0.00 (0.67)	-0.00 (0.92)	-.00 (0.72)	0.00 (0.83)	0.01 (0.59)
Cyanogen in flour (ppm)									
Overall	-0.08 (0.10)	-0.02 (0.70)	**-0.09 (0.01)**	-0.01 (0.72)	**-0.06 (0.04)**	-0.02 (0.52)	**-.04 (0.04)**	**-.09 (0.03)**	1
HH with konzo	-0.01 (0.84)	0.04 (0.62)	-0.07 (0.12)	0.03 (0.50)	-0.02 (0.61)	0.00 (0.94)	-.04 (0.11)	**-.13 (0.03)**	1
HH without konzo	**-0.19 (0.01)**	-0.07 (0.72)	-0.11 (0.09)	-0.07 (0.21)	**-0.14 (0.00)**	-0.06 (0.21)	-.04 (0.13)	-.04 (0.56)	1
Number of nights of cassava soaking									
Overall	-1.20 (0.77)	0.30 (0.94)	-4.95 (0.17)	1.47 (0.61)	-0.97 (0.72)	1.47 (0.56)	-1.23 (0.37)	1.88 (0.62)	12.7 (0.11)
HH with konzo	4.17 (0.48)	0.86 (0.88)	0.07 (0.98)	6.15 (0.07)	-0.90 (0.83)	3.14 (0.35)	-2.45 (0.23)	-3.30 (0.54)	20.23 (0.08)
HH without konzo	-6.97 (0.23)	0.44 (0.93)	-10.41 (0.08)	-3.59 (0.43)	-0.94 (0.78)	-0.42 (0.91)	-0.33 (0.86)	6.47 (0.21)	4.44(0.69)

HH: household; Konzo: irreversible upper-motor disease linked to cyanide neurotoxicity from poorly processed cassava; HSCL-10: Hopkins Symptom Check list-10, GDAS: Goldberg Depression Anxiety Scale; PDQ: Psychomotor developmental quotient by Gensini Gavito, CDA: Child developmental age in months by Gensini Gavito, CF: cyanogen in household flour, ELC: Early Learning Composite, GM: Gross motor, FM: Fine motor

VR: Visual reception, RL: Receptive language, ELA: Expressive language, ppm: Parts per million. Significant p-values are presented in bold.

^#^*p*-value = 0.056.

We did not find an association between the number of nights of soaking cassava roots and the level of cyanogen in cassava flour or the child’s neurodevelopment (*p*-value > 0·05). In the multivariable analyses adjusted for household status, age, gender, dietary cyanogen exposure, child’s anthropometry, parental education, solid food introduction, and wealth index, only child’s linear growth (height-for-age *z*-scores) and content of cyanogen in cassava flour were important predictors of GGS and MSEL (Table 6).

**Table 6:** Overall and by household groups crude coefficient (*p*-value) from multivariable linear regression and GLM analysis Gamma function (Italic) for scores derived from Mullen Scale of Early Learning and Gensini Gavito Scale for the 114 studied children aged 12–48 months in Kahemba, Democratic Republic of Congo.

	ELC	GM	*FM*	*VR*	RL	*ELA*	CDA	PDQ
Age (months)								
Overall	0.53 (0.20)	-.29 (0.54)	0.17 (0.63)	0.18 (0.57)	0.22 (0.37)	0.45 (0.09)	0.69 (0.00)	-.60 (0.08)
HH with konzo	0.29 (0.62)	-.38 (0.70)	-.19 (0.68)	0.03 (0.95)	0.12 (0.77)	0.36 (0.37)	0.95 (0.00)	0.13 (0.77)
HH without konzo	0.51 (0.49)	-.21 (0.75)	0.52 (0.49)	0.16 (0.84)	0.02 (0.97)	0.39 (0.36)	0.46 (0.03)	-.97 (0.12)
Gender								
Overall	2.29 (0.50)	0.69 (0.81)	-.15 (0.96)	0.14 (0.96)	0.64 (0.76)	2.92 (0.19)	0.00 (0.99)	1.55 (0.62)
HH with konzo	-4.54 (0.42)	-.74 (0.89)	-2.63 (0.55)	-2.75 (0.50)	-1.16 (0.76)	3.39 (0.33)	0.82 (0.57)	2.52 (0.58)
HH without konzo	6.96 (0.16)	4.08 (0.35)	5.24 (0.38)	2.81 (0.58)	2.71 (0.29)	1.18 (0.71)	-.10 (0.95)	3.45 (0.51)
Height-for-age *z* scores								
Overall	**7.81 (0.00)**	**3.62 (0.01)**	**3.79 (0.01)**	**5.08 (0.00)**	**4.23 (0.00)**	**2.83 (0.01)**	**1.44 (0.01)**	**4.25 (0.01)**
HH with konzo	**7.53 (0.01**)	-.14 (0.96)	2.91 (0.21)	**5.11 (0.00)**	**5.01 (0.01)**	**4.39 (0.01)**	**2.57 (0.00)**	**8.22 (0.00)**
HH without konzo	4.04 (0.10)	4.43 **(0.04)**	3.59 (0.13)	3.44 (0.15)	1.69 (0.18)	0.39 (0.78)	0.65 (0.43)	1.01 (0.69)
Weight-for-age *z* scores								
Overall	-.35 (0.82)	1.35 (0.29)	-.12 (0.93)	-1.24 (0.33)	0.97 (0.32)	0.35 (0.73)	0.64 (0.15)	1.91 (0.17)
HH with konzo	-1.12 (0.68)	4.53 (0.12)	-.26 (0.92)	-1.90 (0.36)	0.20 (0.91)	-.51 (0.77)	0.37 (0.59)	1.08 (0.62)
HH without konzo	1.81 (0.37)	0.28 (0.89)	0.43 (0.83)	0.49 (0.80)	**2.32 (0.04)**	1.87 (0.13)	0.99 (0.14)	3.52 (0.09)
Child Urinary SCN (μmole/liter)								
Overall	-.00 (0.19)	0.00 (0.91)	-.00 (0.32)	-.00 (0.47)	-.00 (0.18)	-.00 (0.67)	-.00 (0.24)	-.00 (0.21)
HH with konzo	-.00 (0.11)	-.00 (0.93)	-.00 (0.09)	-.00 (0.40)	-.00 (0.08)	-.00 (0.56)	-.00 (0.28)	-.00 (0.24)
HH without konzo	-.00 (0.58)	0.00 (0.49)	0.00 (0.52)	0.00 (0.64)	0.00 (0.59)	-.00 (0.68)	-.00 (0.62)	-.00 (.82)
Cassava Cyanogen (ppm)								
Overall	-.05 (0.34)	-.03 (0.46)	**-.07 (0.03**)	-.00 (0.83)	-.03 (0.25)	-.00 (0.93)	**-.03 (0.02)**	-.07 (0.07)
HH with konzo	0.04 (0.57)	0.04 (0.65)	-.03 (0.43)	0.03 (0.51)	0.01 (0.85)	0.00 (0.96)	**-.05 (0.00)**	**-.15 (0.01)**
HH without konzo	-.08 (0.29)	-.03 (0.62)	-.06 (0.49)	0.04 (0.54)	-.07 (0.07)	-.03 (0.52)	-.02 (0.31)	-.02 (0.76)
Introduction of solid food <6 months								
Overall	6.46 (0.61)	1.27 (0.67)	5.46 (0.08)	4.19 (0.16)	**4.87 (0.03)**	2.59 (0.24)	0.70 (0.48)	2.99 (0.34)
HH with konzo	-2.12 (0.72)	-.58 (0.93)	0.73 (0.89)	-1.12 (0.81)	2.91 (0.46)	0.56 (0.89)	1.39 (0.37)	3.86 (0.43)
HH without konzo	**9.10 (0.06)**	-.85 (0.84)	5.68 (0.22)	8.39 (0.08)	4.61 (0.07)	4.82 (0.08)	0.36 (0.82)	2.31 (0.63)
Father’s education								
Overall	2.36 (0.31)	-.62 (0.75)	1.35 (0.55)	-.60 (0.75)	2.27 (0.12)	1.84 (0.23)	-.47 (0.49)	-.71 (0.73)
HH with konzo	**10.51 (0.02**)	5.08 (0.35)	5.25 (0.17)	3.31 (0.29)	5.55 (0.07)	4.19 (0.14)	-1.61 (0.18)	-4.61 (0.22)
HH without konzo	-.59 (0.82)	-2.35 (0.31)	0.42 (0.89)	-1.87 (0.48)	0.62 (0.65)	0.85 (0.61)	-.62 (0.50)	-1.06 (0.71)
Mother’s education								
Overall	1.57 (0.53)	1.69 (0.41)	-.52 (0.82)	-.62 (0.76)	0.77 (0.62)	1.52 (0.36)	0.44 (0.53)	0.71 (0.74)
HH with konzo	-1.13 (0.81)	0.35 (0.94)	-2.57 (0.49)	-2.71 (0.48)	-.26 (0.93)	-.32 (0.92)	0.10 (0.92)	-.70 (0.83)
HH without konzo	-.03 (0.99)	0.27 (0.92)	-.28 (0.94)	-1.47 (0.66)	-.62 (0.70)	2.19 (0.28)	0.69 (0.51)	1.21 (0.71)
Wealth index (scores)								
Overall	-1.36 (0.07)	-.28 (0.65)	**-1.25 (0.05)**	0.03 (0.96)	-.48 (0.30)	-.46 (0.37)	0.05 (0.81)	-.06 (0.92)
HH with konzo	-2.33 (0.19)	-2.53 (0.25)	-1.68 (0.20)	0.06 (0.96)	-.78 (0.52)	-1.39 (0.22)	-.17 (0.73)	-.89 (0.56)
HH without konzo	-.59 (0.47)	0.05 (0.94)	-1.18 (0.19)	0.34 (0.69)	0.08 (0.86)	-.04 (0.94)	0.07 (0.82)	0.15 (0.86)
Monthly income								
Overall	0.00 (0.19)	0.00 (0.14)	**0.00 (0.02)**	0.00 (0.17)	-.00 (0.45)	0.00 (0.33)	-1.29 (0.91)	-.00 (0.74)
HH with konzo	0.00 (0.66)	0.00 (0.87)	0.00 (0.24)	0.00 (0.25)	-.00 (0.68)	0.00 (0.57)	0.00 (0.48)	0.00 (0.37)
HH without konzo	0.00 (0.29)	0.00 (0.15)	0.00 (0.16)	0.00 (0.71)	-.00 (0.36)	0.00 (0.59)	1.73 (0.92)	-.00 (0.79)

Multivariable analysis included age and gender as adjusted variables. Predictors were gradually entered based on biological and biochemical findings from the univariable linear regression analysis. After adjusting for each predictor, only height-for-age scores remained the significant predictor of early child development, cognition, and motor proficiency, whereas cyanogen content in cassava flour was the main significant predictor of fine motor, and early child development. Significant results are presented in bold.

HH: household, PDQ: Psychomotor developmental quotient by Gensini Gavito, CDA: Child developmental age in months by Gensini Gavito, CF: cyanogen in household flour, ELC: Early Learning Composite, GM: Gross motor, FM: Fine motor, VR: Visual reception, RL: Receptive language, ELA: Expressive language, SCN: thiocyanate, ppm: parts per million. Significant *p*-values are presented in bold.

## Discussion

Our work is the first to report an association between dietary cyanogen exposure, early child neurodevelopment and neurocognition in an area of the DRC affected by konzo, a foodborne disease linked to the chronic consumption of poorly processed cassava has been reported. After adjusting for potential confounders, such as age, gender, and weaning age, we found that child linear growth and cassava cyanogen were the main predictors of early child development and cognition. These two predictors showed consistent significant association with early child development and cognitive outcome with both the MSEL and GGS. At the MSEL, fine motor function was also significantly associated with wealth index and monthly income, whereas the father’s level of education was associated with the early learning composite (general cognitive factor). These aforementioned findings suggest that cassava cyanogen, child’s linear growth, and father’s level of education, are important predictors of early child neurodevelopment and neurocognition in settings relying on the chronic consumption of poorly processed bitter cassava. These factors have been reported in earlier studies in sub-Saharan African studies [18, 36], and are suggestive of social challenges such as poverty and malnutrition (stunting) in households affected by konzo. This is in line with the *Lancet* series on child development that have identified poverty and stunting as major predictors of poor child development in low-income countries [37]. Although the disease konzo is linked to the consumption of poorly processed cassava, the disease is also symptomatic of extreme poverty and linked to other risk factors for early childhood development (e.g., malnutrition, poor parental education). The disease probably worsens the economic situation for the affected households, and may explain some of the associations reported. It is, however, difficult, with our cross-sectional design, and the lack of comparative group from unaffected areas, to speculate about cause-effect relationship.

When looking at early child development and neurocognition per household status (affected vs. unaffected), no significant difference was noticed between the two groups, though children in affected households showed a slight tendency toward lower motor development. The lower motor development was also observed during the clinical assessment of the child’s psychomotor milestones acquisition. This was consistent with previously reported impaired motor proficiencies in older children based on the neurological exam [12]. Although no neurodevelopmental difference was noticed between the two groups of children, their mean level of dietary cyanogen content (> 50 ppm) and urinary thiocyanate (> 600 μmole/litre) were above the safe level of 10 ppm [35] and 350 μmole/litre [15], respectively. This is a plausible explanation for the significant risk of poor early child development in the studied population. While levels of cyanogen in cassava flour may reflect patterns of chronic exposure routinely seen in konzo-affected areas, urinary levels of thiocyanate only reflect exposure in recent days (< 48 hours) [38] and may not be a good marker of subacute or chronic toxicity. This may explain the apparent discrepancy concerning the association between neurodevelopmental deficits and levels of cassava cyanogen, but not with urinary thiocyanate. There is a need for biomarkers that are sensitive and specific enough to measure cumulative past exposure over time. One could argue that the higher level of cyanogen exposure may possibly result from consumption of cassava leaves or smoking. Smoking was ruled out by collecting information on maternal smoking history. Tobacco smoking is generally rare among women in DRC, and as expected none of the selected mothers reported current smoking, whereas only one mother reported smoking during pregnancy. Exposure from cassava leave is unlikely or minimal because leaves are detoxified up to 99% during boiling and cooking process before consumption [39]. There is however a limitation to the dietary cyanogen exposure measurement, which was concomitant to the child assessment, and may or not reflect earlier or cumulative exposure that should have also been assessed. Although there is room for limitation in the present study, the chosen design was our best appropriate choice to primarily study the relationship between dietary cyanogen exposure, and early child neurodevelopment in a resource-limited and challenging setting. A control group from non-konzo areas and non- or low-exposed population would also enhance our understandings of the neurodevelopmental effects associated with cassava cyanogenic exposure.

Furthermore, though children in households affected by the disease seemed similar to those in unaffected households in terms of early child development and neurocognition, some differences were observed relating to their sociodemographic background. Children in affected households were weaned earlier, had lower wealth index scores, higher scores of maternal depressive symptoms, and lower maternal weight than children from unaffected households. Again, these sociodemographic challenges support the disease as symptomatic of poverty and a risk for poor early child development and neurocognition.

Surprisingly, beside dietary cyanogen exposure, and all the above-mentioned important predictors associated with early child development and neurocognition, no association was found with maternal depression, home environment and parenting style. Maternal depressive symptoms were, however, highly reported in konzo-affected households. Nevertheless, our finding should be interpreted with caution, as we did not perform a comprehensive assessment of the mother-child interaction. The cultural context that may imply interactions between the child and his/her extended family, which could challenge an objective assessment of the mother-child interaction. Despite these potential limitations, MSEL has been sensitive to the effects of caregiving quality on the cognitive development of very young children in impoverished Ugandan rural communities affected by HIV [40–42].

Quantifying early child development remains a challenge in low-income countries due to the lack of validated measures of child development [37]. We therefore selected the MSEL, a comprehensive and easy to administer child developmental tool, together with a locally validated tool (GGS) to overcome the challenge. We found a significant positive correlation between the two tools, which supports a previous finding from Africa suggesting that MSEL is a useful child developmental assessment tool for low-income settings [18]. In addition, we conducted a systemic neurological screening examination of all children in order to assess their neurodevelopment and screen for neurological signs of the disease. Unexpectedly, we were able to identify three children with neurological signs suggestive of the disease even though neurological signs have not been described previously. Our finding suggests a relationship between early neurodevelopmental impairments and a milder form of the disease at an earlier age that requires further longitudinal investigations.

A number of limitations should, however, be considered in the interpretation of the findings in the present study. The potential for confounders, selection and misclassification bias may have accounted for these results. The study design and the absence of a control group from unaffected areas do not allow us to determine causation. Eligible households were not randomly selected, and are not representative of the general population. Nonetheless, households were selected from the incident case surveillance list of the health district, to ensure definition validity of eligible households and minimize bias. Finally, due to time and budget constraints, we were unable to conduct a home observation for the comprehensive assessment of the child’s social environment and emotional interaction. We relied on parenting information obtained from parents/caregivers during the home visit interview. Considering that our primary concern was to explore the predictors of early child neurodevelopment in konzo-affected areas, these limitations do not trivialize the importance of the study. The study enabled us to generate useful hypotheses for preventive measures, further prospective and interventional studies on child development in settings exposed to dietary cyanogen.

Despite its weaknesses, the study has valuable strengths that lie on the best available provided evidence of relationship between early child neurodevelopment and dietary cyanogen exposure. We believe our results are important for research on cassava neurotoxicity and early child neurodevelopment. They will contribute to a better understanding of dietary cyanogen neurotoxicity, and child neurodevelopment in sub-Saharan Africa, where research and available data on the topic are scarce. Our preliminary findings constitute the basis for our next randomized intervention trial to establish causality, using wetting method intervention [43] to reduce dietary cyanide exposure, with subsequent benefits to very young children in these households.

## Conclusion

Dietary cyanogen exposure is associated with early child neurodevelopment and neurocognition, even in the absence of clinically evident neurological signs or paralysis. Community-wide interventions for safer food preparation such as the wetting method training/promotion,[15] longer reliance on breastfeeding, and improved nutrition are needed to ensure optimal development in exposed children. There is also a need to promote child development through enhanced maternal mental health, parental contribution and involvement in the child’s rearing to promote opportunities for early learning and cognitive stimulation in impoverished communities.

## Supporting information

S1 Table Description of assessment tools. This table is a description of tools used to assess early child development, neuropsychological outcomes, and maternal depression-anxiety symptoms of the 114 studied children aged 12–48 months and their mothers in Kahemba, Democratic Republic of Congo.
(DOCX)

S1 File STROBE checklist. (DOC)

## Abbreviations

MSEL: Mullen Scale of Early Learning
GGS: Gensini Gavito Scale
PDQ: Psychomotor Developmental Quotient
CDA: Child Developmental Age
HSCL-10: Hopkins Symptoms Checklist-10
GDAS: Goldberg Depression Anxiety Scale
SCN: Thiocyanate
TQQ: Ten Questions Questionnaire
WHO: World Health Organization
GLM: Generalized linear models
SD: Standard deviation
IQR: Inter Quartile Range
PPM: Parts per million
CF: Cyanogen in household flour
ELC: Early Learning Composite
GM: Gross motor
FM: Fine motor
VR: Visual reception
RL: Receptive language
ELA: Expressive language
FBD: Foodborne disease
