# Supplementary material for: Dietary cyanogen exposure and early child neurodevelopment: An observational study from the Democratic Republic of Congo
Source: PLoS One. 2018 Apr 17;13(4):e0193261. doi: 10.1371/journal.pone.0193261 (PMC5903613; doi:10.1371/journal.pone.0193261)
Supplement: S1 Table — This table is a description of tools used to assess early child development, neuropsychological outcomes, and maternal depression-anxiety symptoms of the 114 studied children aged 12–48 months and their mothers in Kahemba, Democratic Republic of Congo. (DOCX) [file pone.0193261.s001.docx]

| **S1 Table. Description of tools used to assess early child development, neuropsychological outcomes, and maternal depression-anxiety symptoms of the 114 studied children aged 12–48 months in Kahemba, Democratic Republic of Congo.** | | |
| --- | --- | --- |
| Tools used | Measures | Target ability/ Function/Scoring |
| Mullen Scales of Early Learning (MSEL) | Childhood development 15 to 60 minutes depending on the child’s age  From birth**–**68 months | Cognitive abilities and motor development  Five Scales: gross motor, visual reception, fine motor skills, expressive and receptive language.  Each scale comprises interactive tasks completed by the child or may be scored through interview of, or with assistance from the parent.  Age, gender-based, and standard scores were obtained using the tables in the manual based on United States of America’s norms.  Scoring range for each test item: from zero to five points.  After scoring all items and computing raw scores, the raw scores are converted into a normative score called the T score for each of the 5 Mullen Scales. T scores from the four cognitive scales (Fine Motor, Visual Reception, Expressive Language, and Receptive Language) are combined into the early learning composite (ELC) score, which provides the general cognitive factor underlying all cognitive performance. |
| Gensini Gavito Scale (GGS) | Psychomotor development | Domains: communication/language, motor function and social adaptation.  Scoring: the scale generates the Psychomotor Development Quotient (PDQ) expressed in percentage = ratio between the Child Developmental Age (CDA) obtained from his/her acquisition in all the three domains at the time of the examination, and his/her chronological age (CA) times 100.  Formula: “PDQ= (CDA/CA) x 100”. |
| Ten Questions Questionnaire (TQQ) | Child development & Disabilities  10 questions | Parents report screening for the child’s perceived developmental disabilities  Administration: interview with caregiver |
| HOME inventory* | Child-mother/caretaker interaction  45 items | Measures favourability of the developmental milieu of the child, the quality, and quantity of interaction that promote learning opportunities. It uses interviews and observation to assess the social, emotional, and cognitive support available to the child in the home setting.  Administration: interview with caregiver and home observation. |
| HOME inventory-18* | Short version  18 items | Parenting style, restriction and punishment, provision of appropriate play materials, opportunities for daily stimulation, and learning. |
| Hopkins symptoms checklist (HSCL-25) | Symptoms of anxiety and depression  25 items | Detect presence and intensity of symptoms of anxiety and depression in the past month  Anxiety (10 items) and depression (15 items)  Tool available in French, and has been translated and adapted for the local language and cultural context.  Scoring of each symptom on a 4-point scale from “not at all” (0) to “extremely” (3). |
| Hopkins symptoms checklist (HSCL-10) | Symptoms of anxiety and depression  10 items | Detect presence and intensity of symptoms of anxiety and depression in the past month  Anxiety and depression (10 items)  Tool available in French, and has been translated and adapted for the local language and cultural context.  Scoring of each symptom on a on a 4-point scale from “not at all” (0) to “extremely” (3). |
| Goldberg Depressive Anxiety Scale (GDAS) | Symptoms of anxiety and depression  18 items | Detect presence and intensity of symptoms of anxiety and depression in the past month. The tool has been validated in the Democratic Republic of Congo.  Anxiety (9 items) and depression (9 items)  Scoring of each symptom on a scale ranging from 0 (not at all) to 5 (extremely) |

* HOME: Home Observation for Measurement of the Environment
