# Supplementary material for: Dietary cyanogen exposure and early child neurodevelopment: An observational study from the Democratic Republic of Congo
Source: PLoS One. 2018 Apr 17;13(4):e0193261. doi: 10.1371/journal.pone.0193261 (PMC5903613; doi:10.1371/journal.pone.0193261)
Supplement: S1 File — (DOC) [file pone.0193261.s002.doc]

S1 File. STROBE Statement-Checklist of items that should be included in reports of ***cross-sectional studies***

|  | Item No | Recommendation |
| --- | --- | --- |
| **Title and abstract** | 1 | (*a*) Indicate the study’s design with a commonly used term in the title or the abstract  **Line 1-2 in manuscript** |
|  |  | (*b*) Provide in the abstract an informative and balanced summary of what was done and what was found  **Line 57 to 92** |
| Introduction | | |
| Background/rationale | 2 | Explain the scientific background and rationale for the investigation being reported  **Line 93 to 121** |
| Objectives | 3 | State specific objectives, including any prespecified hypotheses  **Line 122 to 124** |
| Methods | | |
| Study design | 4 | Present key elements of study design early in the paper  **Line 141 to 149** |
| Setting | 5 | Describe the setting, locations, and relevant dates, including periods of recruitment, exposure, follow-up, and data collection  **Line 128 to 140** |
| Participants | 6 | (*a*) Give the eligibility criteria, and the sources and methods of selection of participants  **Line 141 to 149** |
| Variables | 7 | Clearly define all outcomes, exposures, predictors, potential confounders, and effect modifiers. Give diagnostic criteria, if applicable  **Under study procedure from line 150 to 214** |
| Data sources/ measurement | 8* | For each variable of interest, give sources of data and details of methods of assessment (measurement). Describe comparability of assessment methods if there is more than one group  **Under study procedure** |
| Bias | 9 | Describe any efforts to address potential sources of bias  **See under statistical analyses** |
| Study size | 10 | Explain how the study size was arrived at  **See under study population** |
| Quantitative variables | 11 | Explain how quantitative variables were handled in the analyses. If applicable, describe which groupings were chosen and why  **See under statistical analyses** |
| Statistical methods  **See paragraph on statistical analyses** | 12 | (*a*) Describe all statistical methods, including those used to control for confounding |
|  |  | (*b*) Describe any methods used to examine subgroups and interactions |
|  |  | (*c*) Explain how missing data were addressed |
|  |  | (*d*) If applicable, describe analytical methods taking account of sampling strategy |
|  |  | (*e*) Describe any sensitivity analyses |
| Results | | |
| Participants  **See under sample characteristics line 246 to 255** | 13* | (a) Report numbers of individuals at each stage of study—eg numbers potentially eligible, examined for eligibility, confirmed eligible, included in the study, completing follow-up, and analysed |
|  |  | (b) Give reasons for non-participation at each stage |
|  |  | (c) Consider use of a flow diagram |
| Descriptive data  **See under sociodemographic section line 256 to 283**  **Tables 1 & 2** | 14* | (a) Give characteristics of study participants (eg demographic, clinical, social) and information on exposures and potential confounders |
|  |  | (b) Indicate number of participants with missing data for each variable of interest |
| Outcome data | 15* | Report numbers of outcome events or summary measures |
| Main results  **From line 285 to 310**  **Tables 3 to 6.** | 16 | (*a*) Give unadjusted estimates and, if applicable, confounder-adjusted estimates and their precision (eg, 95% confidence interval). Make clear which confounders were adjusted for and why they were included |
|  |  | (*b*) Report category boundaries when continuous variables were categorized |
|  |  | (*c*) If relevant, consider translating estimates of relative risk into absolute risk for a meaningful time period |
| Other analyses | 17 | Report other analyses done—eg analyses of subgroups and interactions, and sensitivity analyses |
| Discussion | | |
| Key results | 18 | Summarise key results with reference to study objectives  **Line 311 to 326** |
| Limitations | 19 | Discuss limitations of the study, taking into account sources of potential bias or imprecision. Discuss both direction and magnitude of any potential bias  **Line 357-364, 377-379, 394-407** |
| Interpretation | 20 | Give a cautious overall interpretation of results considering objectives, limitations, multiplicity of analyses, results from similar studies, and other relevant evidence  **See under paragraph of discussion** |
| Generalisability | 21 | Discuss the generalisability (external validity) of the study results  **See under paragraph of discussion** |
| Other information | | |
| Funding | 22 | Give the source of funding and the role of the funders for the present study and, if applicable, for the original study on which the present article is based  **Under Acknowledgment.** |

*Give information separately for exposed and unexposed groups.

**Note:** An Explanation and Elaboration article discusses each checklist item and gives methodological background and published examples of transparent reporting. The STROBE checklist is best used in conjunction with this article (freely available on the Web sites of PLoS Medicine at http://www.plosmedicine.org/, Annals of Internal Medicine at http://www.annals.org/, and Epidemiology at http://www.epidem.com/). Information on the STROBE Initiative is available at www.strobe-statement.org.
